# What causes wound pain?

**DOI:** 10.1186/1757-1146-4-S1-P22

**Published:** 2011-05-20

**Authors:** Nikki Frescos

**Affiliations:** 1 Department of Podiatry, La Trobe University, Bundoora, Victoria, 3086, Australia

## 

Chronic leg wounds have a considerable impact on quality of life. It has been reported that over 70% of patients experience pain associated with their wounds which can range from moderate to severe. Wound pain and intensity is highly variable, it is not an accurate predicator to make clinical assumptions that specific wound types or wound size will define the type of pain the patient is experiencing. Pain intensity can be stable over time, vary day-to-day, and may increase. Wound pain is an indicator of ineffective wound management, the underlying causal pathology has not being identified nor treated or infection is present. Ineffective wound pain management can result in delayed healing and lack of compliance by the patient. Wound pain can be caused by skin damage, nerve damage, blood vessel injury, infection and ischaemia. It can lead to hypoxia which impairs wound healing and increase infection rates. As tissue oxygen decreases, there is a decrease in the production of leucocytes which provides opportunity for bacteria to colonise the wound thus leading to infection Nerve damage is constantly occurring in the wound, due to the biochemical processes occurring in the wound and external stimuli such as wound debridement, cleansing or dressing changes. It is normally the underlying pathology or aetiology of chronic wounds which dictates the sort of pain that the patient may experience. In order to provide appropriate and effective treatment for addressing wound pain it is necessary to understand the aetiology of the wound, treat the underlying cause and eliminate the noxious stimuli. This presentation will provide an overview of common leg wound aetiologies and related pain symptoms.

